# Inhibition of BET bromodomain proteins as a therapeutic approach in prostate cancer

**DOI:** 10.18632/oncotarget.1572

**Published:** 2013-11-23

**Authors:** Anastasia Wyce, Yan Degenhardt, Yuchen Bai, BaoChau Le, Susan Korenchuk, Ming-Chih Crouthamel, Charles F. McHugh, Robert Vessella, Caretha L. Creasy, Peter J. Tummino, Olena Barbash

**Affiliations:** ^1^ Cancer Epigenetics DPU, Oncology R&D, GlaxoSmithKline, Collegeville, PA, USA; ^2^ Molecular Medicine Unit, Oncology R&D, GlaxoSmithKline, Collegeville, PA, USA; ^3^ Departments of Urology and Microbiology, University of Washington School of Medicine, Seattle, WA, USA

**Keywords:** BET, Brd4, c-Myc, prostate cancer, bromodomain

## Abstract

BET (bromodomain and extra-terminal) proteins regulate gene expression through their ability to bind to acetylated chromatin and subsequently activate RNA PolII-driven transcriptional elongation. Small molecule BET inhibitors prevent binding of BET proteins to acetylated histones and inhibit transcriptional activation of BET target genes. BET inhibitors attenuate cell growth and survival in several hematologic cancer models, partially through the down-regulation of the critical oncogene, *MYC*. We hypothesized that BET inhibitors will regulate *MYC* expression in solid tumors that frequently over-express *MYC*. Here we describe the effects of the highly specific BET inhibitor, I-BET762, on *MYC* expression in prostate cancer models. I-BET762 potently reduced *MYC* expression in prostate cancer cell lines and a patient-derived tumor model with subsequent inhibition of cell growth and reduction of tumor burden *in vivo*. Our data suggests that I-BET762 effects are partially driven by *MYC* down-regulation and underlines the critical importance of additional mechanisms of I-BET762 induced phenotypes.

## INTRODUCTION

Cancer epigenetics is a rapidly progressing field of oncology that has recently demonstrated the use of epigenetic drugs as targeted treatments in preclinical models of several cancer types, such as EZH2 inhibitors in B-cell lymphomas and rhabdoid tumors and DOT1L inhibitors in acute myeloid leukemia [1-3]. Recent studies demonstrated that small molecule inhibitors of BET proteins attenuate cellular proliferation and survival in several preclinical tumor models, including hematological and solid cancers. The BET protein family includes four members: BRD2, BRD3, BRD4 and BRDT. These proteins function as readers of acetylated chromatin that translate chromatin status into activated transcription through RNA PolII regulation. BET proteins play a role in the cellular proliferation through cell cycle and apoptosis regulation [4]. GSK525762A (I-BET762) is a specific and potent inhibitor of BET protein binding to acetylated histones [5,6]. BET inhibitors are efficacious in models of NUT midline carcinoma (NMC), a rare epithelial tumor type driven by the fusions of NUT protein with either BRD3 or BRD4 genes [7]. Additionally, BET inhibitors have shown therapeutic potential in preclinical models of multiple myeloma and acute myeloid leukemia through multiple mechanisms including down-regulation of *MYC* expression [8-13].

Amplification of the *MYC* locus is frequent in prostate cancer (30%) and is associated with disease progression and Gleason score [14]. c-Myc protein levels are also elevated in prostate tumors compared to normal prostate epithelium [15], with a stepwise increase from low-grade to high-grade prostatic intraepithelial neoplasia (PIN). *MYC* overexpression is sufficient to transform benign human prostate epithelium *in vitro* [16] and *MYC* transgenic mice develop PIN [17], suggesting that *MYC* plays a role in prostate cancer initiation. *MYC* is a promising target in prostate cancer as demonstrated in the preclinical models of prostate cancer with antisense nucleotides [18].

We have sought to explore *MYC* regulation in prostate carcinoma models in the response to I-BET762 treatment. We observed that I-BET762 treatment inhibits *MYC* expression accompanied by the growth inhibition and decreased survival in prostate cancer cells that over-express *MYC*. Importantly, BET inhibition reduces tumor burden in a primary model of castration resistant prostate cancer that expresses high levels of *MYC*. Our data suggest that BET inhibitors offer new therapeutic approach to treat prostate tumors driven by *MYC*.

## RESULTS

### I-BET762 Inhibits Cell Growth and Induces Cell Death in a Subset of Prostate Cancer Cell Lines

**Figure 1:**
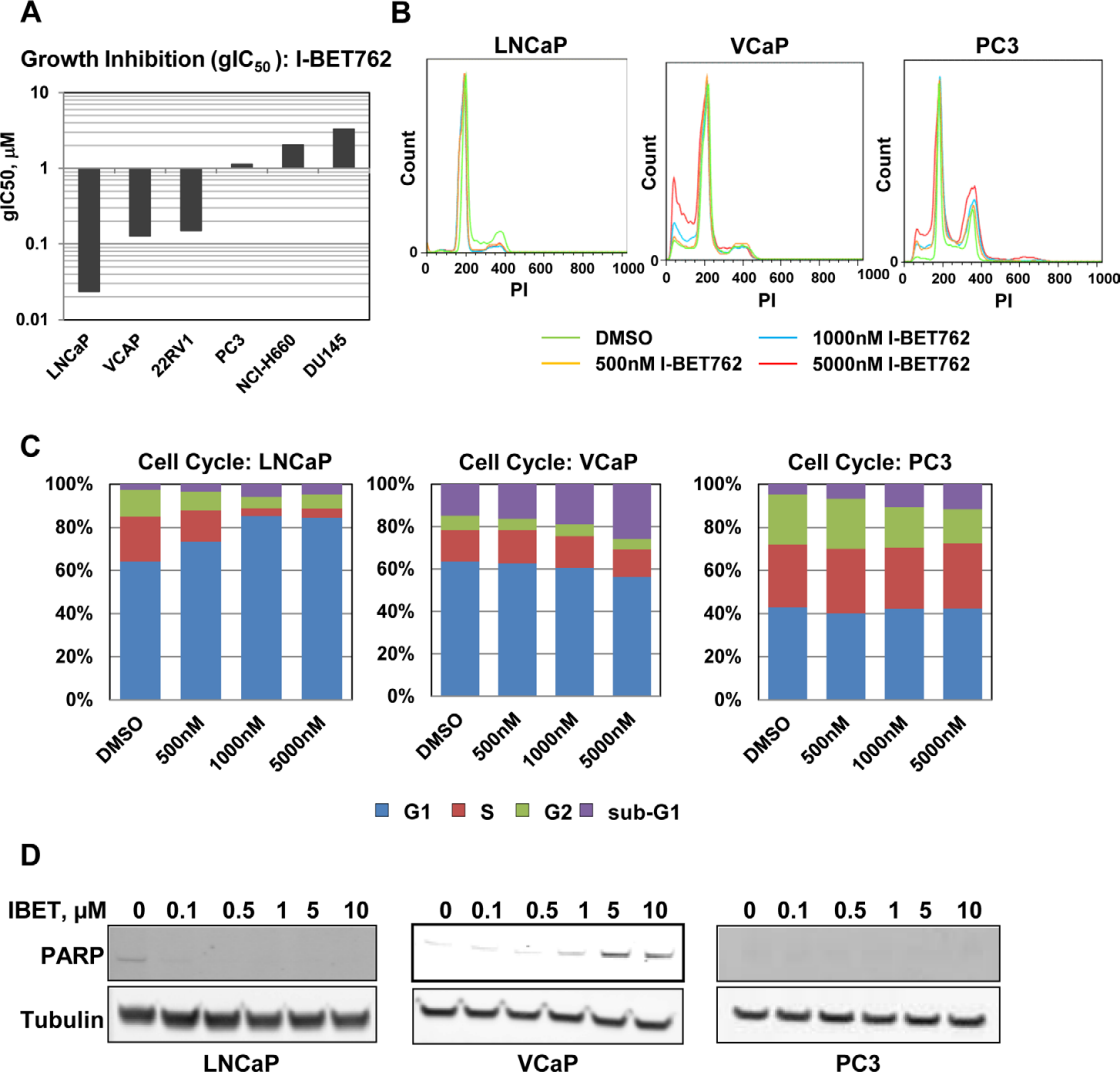
Characterization of I-BET762 sensitivity in prostate cancer cell lines A, Average growth IC_50_ (gIC_50_) values observed for I-BET762 in a panel of prostate cancer cell lines obtained from a 6 day growth-death assay (minimum n=2). B, Histograms generated from cell cycle analysis in the indicated cell lines following 6 days treatment with vehicle, 0.5 µM, 1 µM, or 5 µM I-BET762. Data shown was from a single experiment representative of typical results. C, Stacked bar graphs representing the average population of cells in various phases of the cell cycle following treatment with I-BET762 for three days in the indicated cell lines (n=2). D, Western blot analysis of cleaved PARP in the indicated cell lines following three day treatment with a titration of I-BET762.

We screened a panel of prostate cancer cell lines for sensitivity to I-BET762 in a 6 day growth assay. While some level of growth inhibition was observed in all cell lines, a subset of the cell line panel was particularly sensitive to I-BET762, with growth IC_50_ (gIC_50_) values ranging from 25 nM to 150 nM (Figure 1A). Cell cycle analysis in a subset of cell lines revealed a range of responses to I-BET762 treatment. Concentration-dependent G1 arrest was observed in the LNCaP cell line, whereas sub-G1 accumulation was detected in VCaP cells (Figure 1B, C; Supplemental Table S1). PC3 cells exhibited minimal growth inhibition in response to I-BET762; however cell cycle analysis revealed a slight increase in the sub-G1 fraction suggesting low-level cell death in response to compound treatment.

PARP cleavage confirmed induction of apoptosis in VCaP cells (Figure 1E). In contrast, we detected minimal cleaved PARP in cell lines that were either less sensitive to I-BET762 (PC3), or exhibited G1 arrest in response to the compound (LNCaP). Our data therefore suggest that growth inhibition and apoptosis are independently triggered in a subset of prostate cancer cell lines in response to BET inhibitor treatment.

To gain an understanding of the individual contributions of BRD2, BRD3, and BRD4 to the growth inhibitory effects observed upon treatment with I-BET762, we transfected LNCaP cells with siRNAs specific for the individual BET proteins and measured effects on cell growth after 6 days (Supplemental Figure S1). While the most consistent and pronounced effects were observed following BRD4 knockdown, loss of BRD2 and BRD3 also inhibited LNCaP cell growth, suggesting that all three BET family proteins contribute to the potent growth responses to BET inhibitors observed in prostate cancer cell lines.

### High AR, c-Myc, and BET Protein Expression Observed in Cell Lines Sensitive to I-BET762

**Figure 2:**
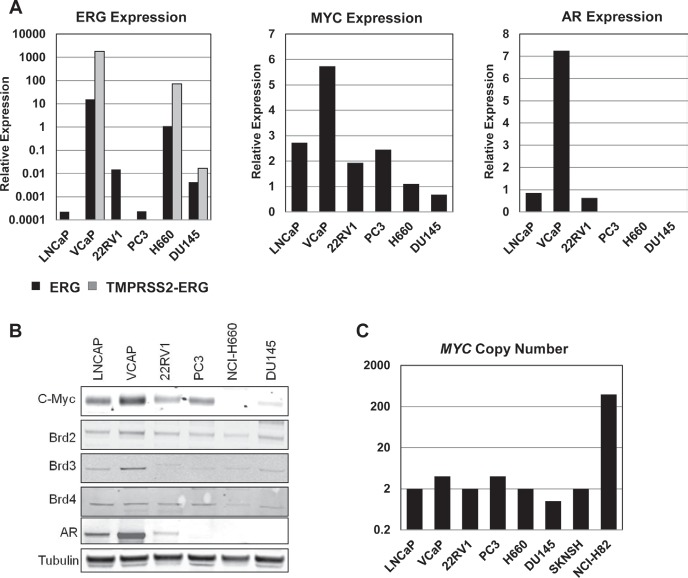
Expression of various driver oncogenes in prostate cancer cell lines A, qPCR analysis of basal *MYC*, *AR*, *ERG*, and *TMPRSS2-ERG* expression in the indicated cell lines. Data were normalized to expression of *GAPDH*, and are presented as relative expression compared to normal prostate RNA. B, Western blot analysis of basal expression of c-Myc, ERG, AR, and BET proteins in the prostate cancer cell line panel. C, Copy number analysis for *MYC* in the prostate cancer cell line panel. SK-N-SH and NCI-H82 were included as controls for normal and amplified *MYC* copy number, respectively.

To gain a better understanding of the mechanisms through which BET inhibitors regulate cell growth and death in prostate cancer cell lines, we analyzed basal expression of BET proteins and several oncogenes that are known drivers in prostate cancer, including *ERG*, *MYC*, and *AR* (Figure 2). *ERG* and *TMPRSS2-ERG* RNA expression was observed in the VCaP and H660 cell lines (Figure 2A), which possess a translocation resulting in a *TMPRSS2-ERG* fusion transcript [19,20]. High c-Myc expression was observed in several cell lines (Figure 2A, B). While we did not observe a significant correlation between *MYC* RNA expression and sensitivity to I-BET762 (Supplemental Figure S2), c-Myc protein expression appeared to qualitatively track with sensitivity, with the highest levels of c-Myc protein occurring in the cell lines with the lowest gIC_50_. Despite the high level of c-Myc expression we did not detect copy number gains at the *MYC* locus in any cell lines (Figure 2C). Similarly, we observe no significant correlation between RNA expression of *AR* or BET family genes and sensitivity (Supplemental Figure S2); however, protein expression, particularly for AR and BRD3, appear to be higher in cell lines exhibiting greater sensitivity to I-BET762 (Figure 2B).

### I-BET762 Down-regulates *MYC* Expression Signatures in a Subset of Prostate Cancer Cell Lines

To gain a better understanding of the pathways regulated by I-BET762 treatment in prostate cancer, we profiled gene expression changes in four cell lines (LNCaP, VCaP, NCI-H660, and PC3) following 24 hours of treatment with I-BET762 via Affymetrix microarrays. Significant down-regulation of genes associated with cell cycle and DNA replication were observed in LNCaP and VCaP (Table 1, Supplemental Table S2), which is consistent with the potent growth inhibition observed in these cell lines. Despite high level expression in VCaP and LNCaP, we observe no change in *AR* expression upon I-BET762 treatment in any of the cell lines profiled (Supplemental Table S3). Consistent with sustained expression of AR, we observe minimal effects on androgen-dependent gene expression or growth in LNCaP cells treated with I-BET762 (Supplemental Figure S3), suggesting that the potent growth inhibition observed in LNCaP cells with I-BET762 is due to perturbation of other pathways. Similarly, despite high level expression of *ERG* transcripts in VCaP and H660 cells, we observe no consistent changes in expression of *ERG*, *TMPRSS2-ERG*, or downstream targets in either cell line (Supplemental Table S3, Supplemental Figure S4).

**Table 1:** Pathways Most Significantly Down-regulated by I-BET762 in LNCaP Cells

Term	Fold Enrichment	p-value
GO:0006260~DNA replication	2.966618045	1.49E-07
GO:0042254~ribosome biogenesis	3.356584772	9.85E-07
REACT_383:DNA Replication	3.281781377	2.6E-06
GO:0006268~DNA unwinding during replication	10.00720926	4.6E-06
hsa03030:DNA replication	5.264214047	7.62E-06
GO:0006259~DNA metabolic process	1.878883924	1.32E-05
GO:0006270~DNA replication initiation	8.756308101	1.39E-05
GO:0032508~DNA duplex unwinding	7.783384979	3.49E-05
GO:0032392~DNA geometric change	7.783384979	3.49E-05
GO:0022613~ribonucleoprotein complex biogenesis	2.459637107	7.4E-05

Top 10 most significantly down-regulated pathways in LNCaP cells treated with 0.5µM I-BET762. Complete functional analyses for LNCaP, VCaP, H660, and PC3 cells are detailed in Supplemental Table S2.

**Figure 3:**
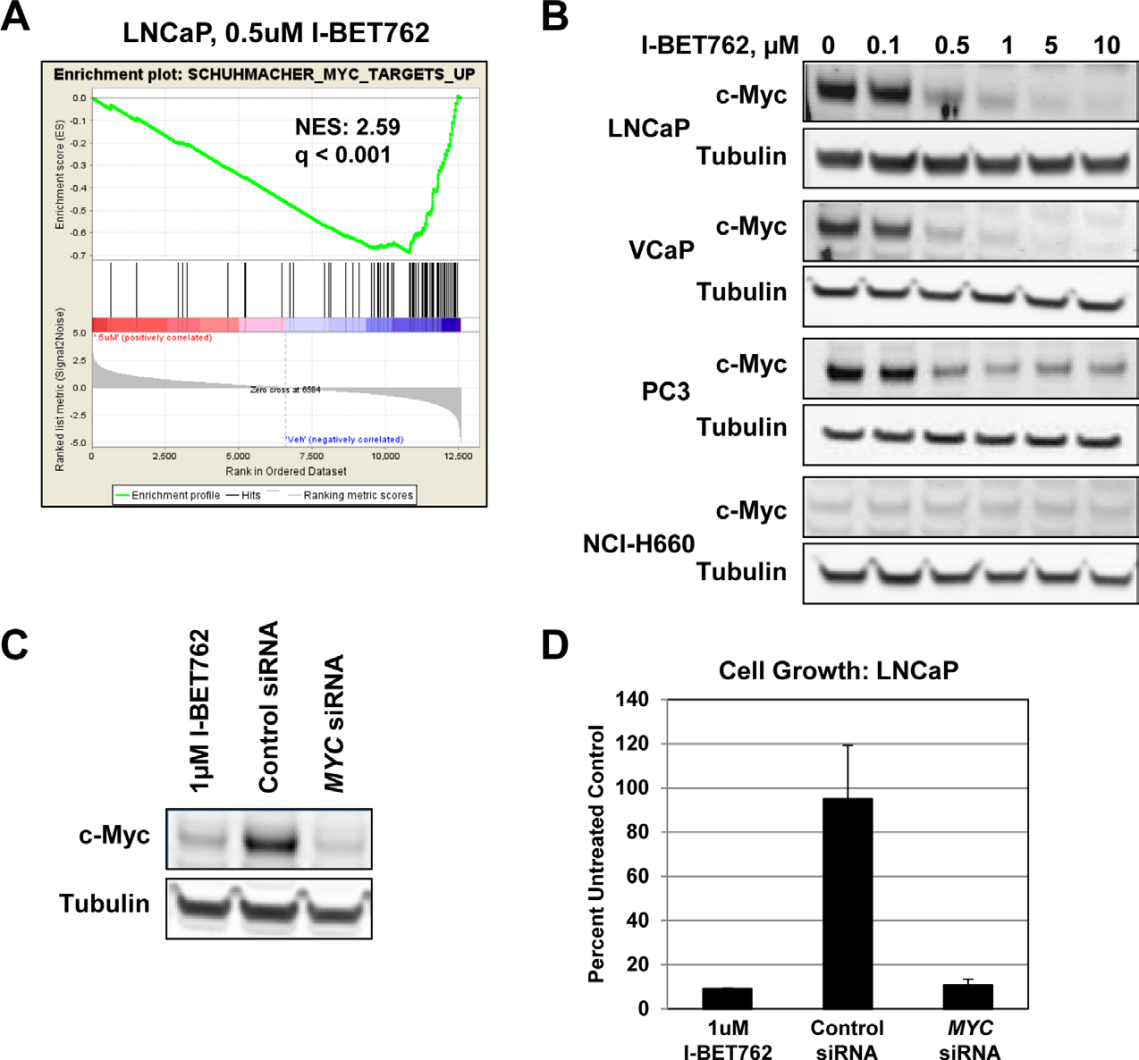
I-BET762 treatment modulates expression of *MYC* and c-Myc-driven pathways in prostate cancer cell lines A, GSEA enrichment plot showing down-regulation of a *MYC* signature in LNCaP cells treated with 0.5 µM I-BET762 for 24 hours. Normalized enrichment score (NES) and FDR q value are indicated. B, Western blot analysis of c-Myc expression in the indicated cell lines following treatment with a titration of I-BET762 for three days. C, Western blot analysis of c-Myc expression following 3 days treatment with 1µM I-BET762, control siRNA, or *MYC* siRNA. D, Analysis of cell proliferation in I-BET762 or siRNA-treated LNCaP cells 6 days post-treatment. Data is presented as percent of untreated control cells, and represents the mean +/− SDM for two independent biological replicates.

Among the genes down-regulated in our data set was *MYC*, which was previously shown to be silenced by BET inhibitors in a number of tumor types [8-11]. GSEA analysis identified *MYC* signatures among the top 10 most significantly down-regulated signatures in the LNCaP cell line following treatment with 0.5 µM I-BET762 (Figure 3A). Additional analysis revealed significant down-regulation of *MYC* signatures in 3 out of the 4 cell lines profiled (Supplemental Figure S5), as well as concentration-dependent suppression of c-Myc expression in these same cell lines (Figure 3B). Suppression of c-Myc and its downstream targets was most pronounced in cells lines exhibiting the greatest sensitivity to I-BET762, suggesting that perturbations in *MYC* pathways may contribute to the growth effects observed in prostate cancer cell lines.

### *MYC* Silencing Contributes to I-BET762 Sensitivity in a Subset of Prostate Cancer Cell Lines

We next examined the consequences of *MYC* down-regulation in the LNCaP cell line using siRNA to knock down *MYC* expression. c-Myc protein expression in the siRNA treated sample was reduced to a similar degree as treatment with 1µM I-BET762 (Figure 3C), and siRNA or compound treatment resulted in similar effects on cell growth (Figure 3D). Targeted inhibition of any single BET protein via siRNA treatment produced minimal effects on c-Myc expression (Supplemental Figure S1), suggesting multiple BET proteins contribute to the silencing of c-Myc expression observed upon treatment with I-BET762.

**Figure 4:**
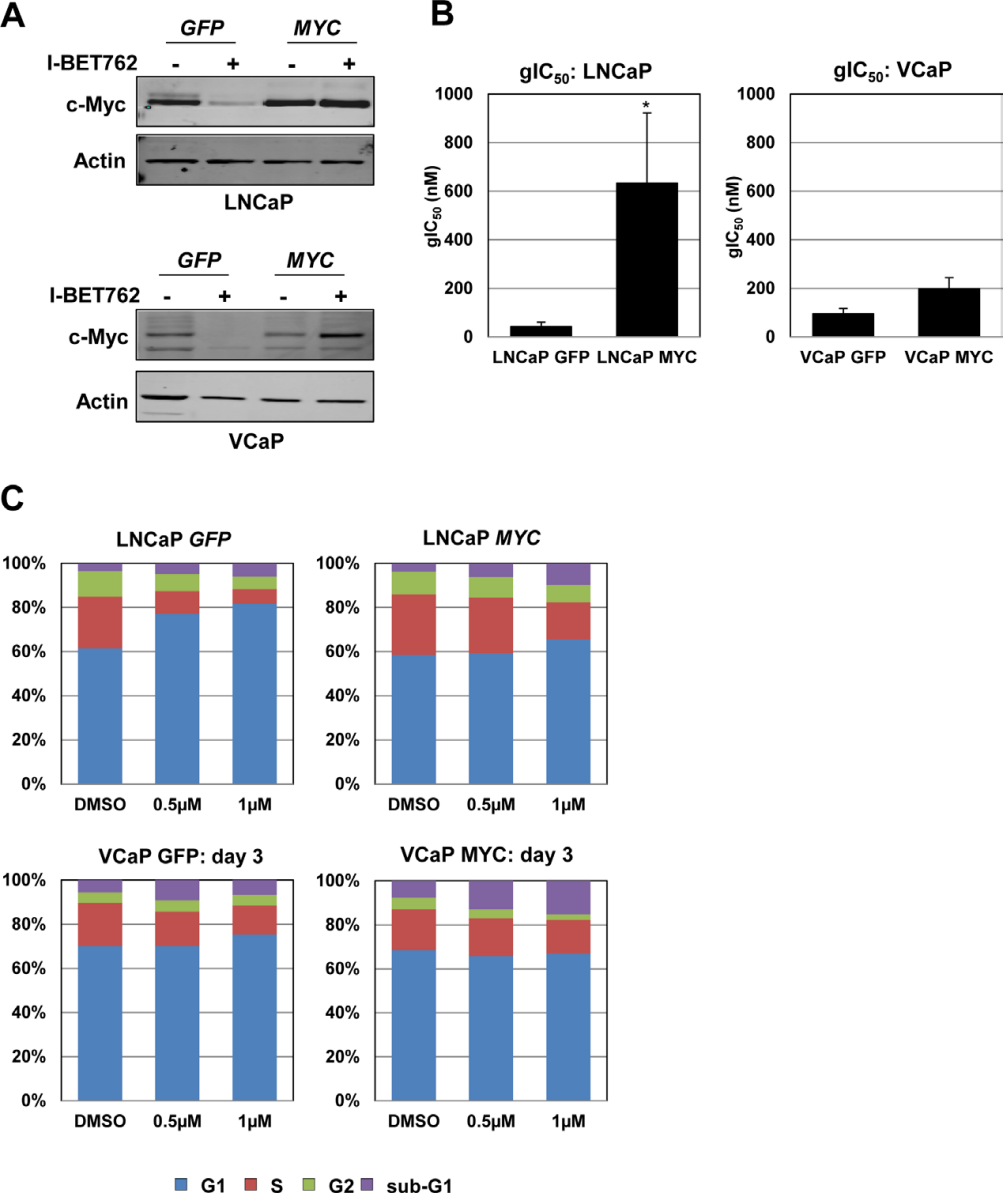
Persistent *MYC* expression partially rescues I-BET762-mediated growth effects in LNCaP cells, but has minimal effect in VCaP cells A, Western blot analysis of c-Myc expression in LNCaP cells (top) and VCaP cells (bottom) overexpressing *GFP* or *MYC* following a three day treatment with 1 µM I-BET762. B, Average gIC_50_ values from a 6 day growth-death assay for LNCaP or VCaP cells overexpressing *GFP* or *MYC* (n=3 for LNCaP; n=2 for VCaP). Asterisk indicates statistical significance as determined by t test (p=0.024). C, Stacked bar graphs representing the average population of cells in various phases of the cell cycle following three day treatment with I-BET762 in LNCaP or VCaP cells overexpressing *GFP* or *MYC* (n=2 for LNCaP; n=3 for VCaP).

To further examine the contribution of *MYC* silencing to the effects observed upon I-BET762 treatment, we ectopically overexpressed *GFP* or *MYC* in LNCaP and VCaP cells using lentiviral expression constructs. Overall levels of c-Myc expression were relatively similar between *GFP* and *MYC* overexpressing cells; however, exogenous c-Myc expression was not down-regulated by I-BET762 treatment in either cell line (Figure 4A). Analysis of growth effects in *GFP* of *MYC* overexpressing LNCaP cells following I-BET762 treatment revealed a statistically significant 15-fold shift in gIC_50_ when *MYC* expression could not be silenced (Figure 4B). Consistent with the shift in gIC_50_, we observed a reduction in G1 arrest in the *MYC* overexpressing cells as determined by cell cycle analysis (Figure 4C). In contrast, ectopic *MYC* expression in VCaP cells produced minimal effects on growth inhibition (Figure 4B, C) or induction of apoptosis in response to the drug treatment (Supplemental Figure S6). Thus, our data indicate that growth inhibition in response to I-BET762 is due, at least in part, to silencing of c-Myc and its downstream pathways in a subset of prostate cancer cell lines.

### I-BET762 Inhibits Growth of Primary Prostate Cancer Xenografts

**Figure 5:**
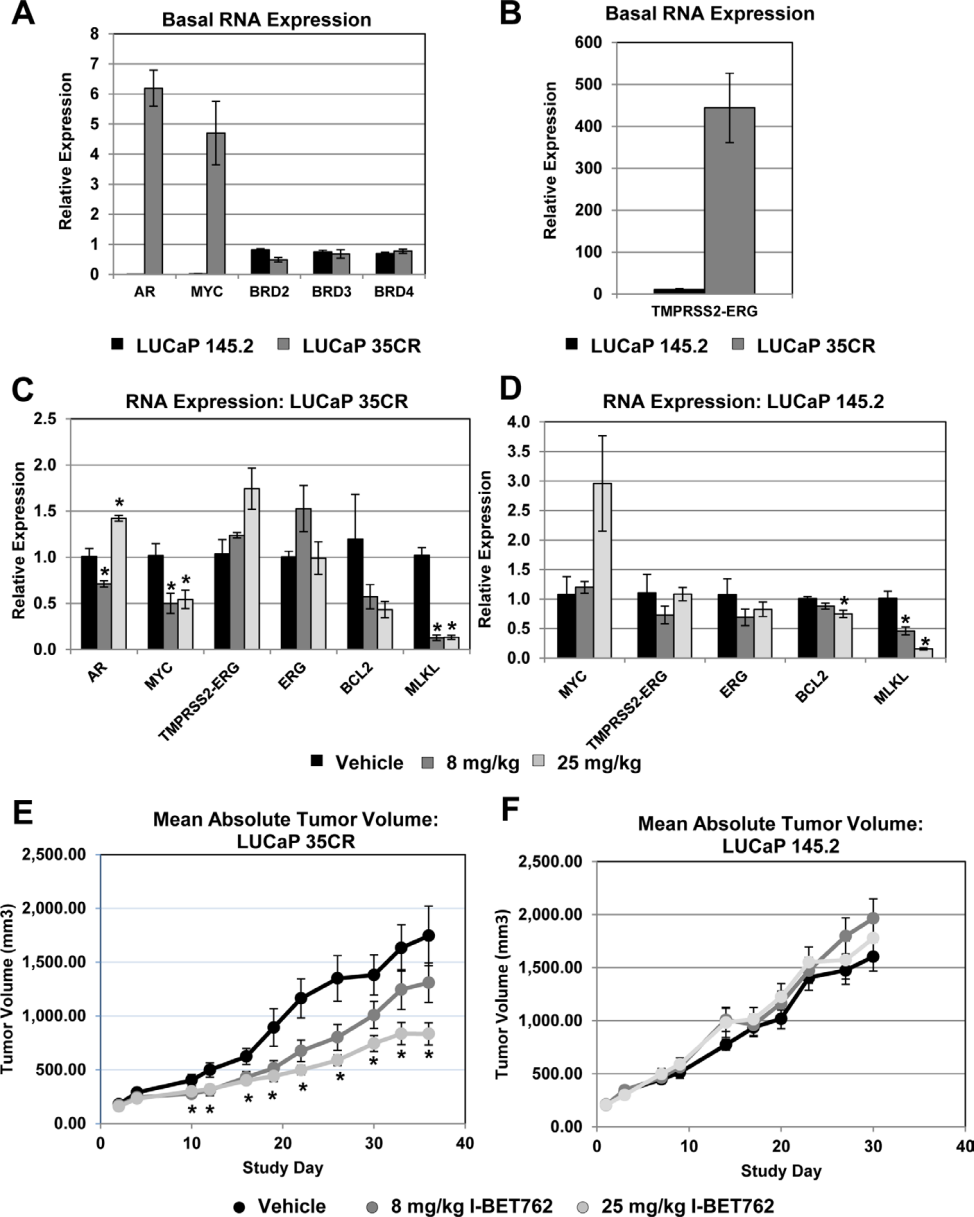
I-BET762 inhibits tumor growth in primary xenograft models with high *MYC*, *AR*, and *TMPRSS2-ERG*expression A, qPCR determination of basal expression of the indicated genes in the LUCaP 35CR and LUCaP 145.2 primary xenograft models. Data were normalized to expression of *GAPDH*, and are presented as relative expression compared to normal prostate RNA. Data represents the mean value ± SEM from three animals. B, qPCR analysis of *TMPRSS2-ERG* expression as described in A. C, qPCR analysis of gene expression changes induced by I-BET762 treatment in the LuCaP 35CR xenograft model. Expression was analyzed in mice treated daily with vehicle, 8 mg/kg I-BET762, or 25 mg/kg I-BET762 for 36 days. Samples were collected 8 hours after dosing on day 36. Data were normalized to expression of *GAPDH*, and are presented as fold induction compared to vehicle treated controls. Data represents the mean ± SEM from three animals. Asterisks indicate significant changes as determined by two-tailed, unpaired t test (p< 0.05). D, qPCR analysis of gene expression changes induced by I-BET762 treatment in the LuCaP 145.2 xenograft model. Expression was analyzed in mice treated daily with vehicle, 8 mg/kg I-BET762, or 25 mg/kg I-BET762 for 30 days. Samples were collected 8 hours after dosing on day 30. Data were analyzed and presented as described in C. E, Mean absolute tumor volumes ± SEM for LUCaP 35CR xenografts following treatment with 8 mg/kg or 25 mg/kg I-BET762. Asterisks indicate p < 0.05 as determined by the Mann-Whitney test. TGI for 8 mg/kg was 27% on Day 36 (n= 10; p= 0.44). TGI for 25 mg/kg was 57% on Day 36 (n=10; p =0.006). F, Mean absolute tumor volumes ± SEM for LUCaP 145.2 xenografts following treatment with 8 mg/kg or 25 mg/kg I-BET762. No significant TGI was observed at either dose.

We next sought to determine if I-BET762 inhibits prostate tumor growth *in vivo*. We characterized *AR*, *MYC*, and *TMRPSS2*-*ERG* expression in two established primary prostate cancer xenograft models: the castration-resistant LuCaP 35CR model (formerly named LuCaP 35V; [21]) and the neuroendocrine prostate cancer model LuCaP 145.2. While the levels of BRD2, BRD3, and BRD4 are comparable between the two models, LuCaP 35CR expresses high levels of *AR*, *MYC*, and *TMPRSS2-ERG*, whereas expression of these genes are low or undetectable in LuCaP 145.2 (Figure 5A, B). Repeated dosing of mice bearing LuCaP 35CR xenografts with I-BET762 resulted in significant down-regulation of *MYC* as well as *MLKL* (Figure 5C), a gene that was down-regulated in several cell lines upon I-BET762 treatment in culture (Supplemental Table S4). In contrast, we observe no significant change in the low-level expression of *MYC* in the LUCaP 145.2 model; although significant changes in *MLKL* and the BET target gene *BCL2* were detected (Figure 5D; [22]). Consistent with our *in vitro* data, I-BET762 produced little to no effect on *ERG*, or *TMPRSS2-ERG* expression in either xenograft model (Figure 5C, 5D). AR expression was undetectable in LUCaP 145.2, and we observed only small, variable effects on AR expression in LuCaP 35CR.

I-BET762 treatment resulted in dose-dependent tumor growth inhibition (TGI) in the LuCaP 35CR model (Figure 5E). The 25 mg/kg treatment group reached a statistically-significant TGI of 57% (n=10, p=0.006). The 8 mg/kg group exhibited a 27% reduction in tumor growth; however, this effect did not reach statistical significance. In contrast, we observe no significant TGI in the LuCaP 145.2 model (Figure 5F). I-BET762 was well-tolerated in both models at all doses tested (Supplemental Figure S7). Our primary prostate xenograft studies therefore confirm the activity we observed for I-BET762 in prostate cancer cell lines, and highlight the potential of BET inhibitors as therapeutic agents in prostate tumors with high *MYC* expression.

## DISCUSSION

In these studies, we demonstrate that BET proteins regulate *MYC* levels in prostate cancer models and the BET inhibitor, I-BET762, potently decreases *MYC* expression in cell lines and a patient-derived model with high *MYC* expression. Our experiments also show that I-BET762 potently inhibits growth in a subset of cell lines and decreases the tumor burden in androgen-refractory primary patient-derived prostate carcinoma (Figure 5), suggesting that I-BET762 might be a treatment option in advanced prostate cancer.

Prostate carcinogenesis is a stepwise process that starts with the prostatic intraepithelial neoplasia (PIN) and progresses to the invasive carcinoma and androgen refractory disease. *MYC* activation is thought to be an early event in prostate cancer [23]. However, targeting *MYC* expression still represents an intervention option in prostate cancer, as demonstrated by the antisense-nucleotide approach in models of prostate cancer, where targeting *MYC* results in the suppression of proliferation and the reduction of prostasphere formation [24].

Our observation that a BET inhibitor silences c-Myc expression and downstream pathways in prostate cancer cell lines is consistent with a previous report [25]. We further show that prostate cancer cell lines with high c-Myc protein expression are more sensitive to I-BET762 (LNCaP and VCaP, Figure 1A) compared to cell lines with low c-Myc protein expression (NCI-H660 and DU145, Figure 1A). In the LNCaP cell line, *MYC* overexpression partially rescued the I-BET762 induced anti-growth phenotype (Figure 4), suggesting that *MYC* significantly contributes to the response to I-BET762. Previously, it has been reported that regulation of *MYC* expression by BET inhibitors in solid tumors is somewhat cancer-type dependent. In glioblastomas (GBM), BET inhibitor treatment decreases *MYC* expression only in a subset of patient-derived models and exogenous *MYC* expression partially rescues BET driven anti-proliferative effects [26]. These results are consistent with observations that *MYC* is critical for self-renewal and differentiation in GBM [27]. Similarly, in neuroblastoma models BET inhibitors down-regulate *MYCN* expression and the regulation of cellular growth by BET is partially *MYCN*-dependent [28,29]. In contrast to these studies, in lung carcinomas, BET inhibition rarely down-regulates *MYC* and the effect of BET inhibitors on proliferation of lung cancer cells is *MYC*-independent [30]. Further understanding of molecular mechanisms that determine the role of BET proteins in *MYC* regulation will help to guide the selection of *MYC*-driven tumor types that might benefit from BET inhibition therapy.

*MYC* overexpression only partially rescued I-BET762 phenotype in LNCaP cells and had minimal effects in VCaP cells, underscoring that additional mechanisms contribute to I-BET762 induced anti-proliferative effects. In cell lines with potent inhibition of cellular growth (LNCaP and VCaP) we observed attenuation of cell cycle pathways, including the down-regulation of MCM genes and genes important for G1/S transition (Table 1, Supplemental Table S2). BRD4 is a known regulator of G1/S transition in mouse fibroblasts, where it activates transcription of Cyclin D, MCM2, ORC2 and other genes that are involved in the cell cycle progression [31]. It is important to note that LNCaP and VCaP cells express higher levels of BRD4 protein and may be dependent on BRD4 to potentiate the G1/S transition. Future studies will be aimed at determining the molecular mechanisms of I-BET762 anti-growth phenotype in the prostate tumor models to identify additional prostate cancer patient populations that might benefit from BET inhibitor treatment.

I-BET762 treatment had minimal effects on expression of AR or its downstream target genes in the LNCaP cell line under non-stimulating conditions (Supplemental Table S3, Supplemental Figure S3). It was previously reported that BRD2 is recruited to the promoters of AR target genes upon androgen stimulation, and that BRD2 recruitment and expression of AR targets are diminished upon treatment with the BET inhibitor JQ1 [25,32]. While we cannot rule out a potential role of BET proteins in regulating expression of androgen-stimulated gene expression programs, our data suggest that perturbation of AR-driven pathways plays a minimal role in cellular response to I-BET762 under normal growth conditions.

In addition to the regulation of cellular growth in prostate cancer models, BET inhibition results in the induction of cell death in some cell lines (VCaP, Figure 1). Surprisingly, our microarray analyses suggest that BET inhibition did not attenuate the expression of genes involved in cell death pathways in the VCaP cell line, but had an effect on the expression of cell death pathway genes in a cell line with a weak, cytostatic response to I-BET762, PC3 (Supplemental Table S2). Therefore, the induction of cell death by I-BET762 in prostate models may be context-dependent and will be investigated in future studies.

## METHODS

### Cell lines and Reagents

Cell lines were obtained from **ATCC and a**uthenticated via STR profiling. All cell lines were grown in RPMI-1640 medium containing 10% FBS, 2 mM GlutaMAX (Life Technologies), and 1 mM sodium pyruvate. For androgen-dependent growth assays, LNCaP cells were cultured in phenol red-free RPMI-1640 containing 10% charcoal-stripped FBS, and 10nM DHT. Antibodies and qPCR primers are listed in Supplemental Methods.

### Cell Line Growth Assay

Cell line growth assays were performed as previously described [29]. Briefly, cells were seeded into 96 or 384-well plates at a density optimized for 6 days of growth. T_0_ measurements were taken the following day using Cell Titer-Glo (Promega) following the manufacturer's instructions. Plates were read on a Safire 2 (Tecan) plate reader. Remaining plates were treated with DMSO or a titration of I-BET762 for 6 days and developed as described above. Results were plotted as a percent of the T_0_ value, (normalized to 100%) versus compound concentration, and a 4-parameter equation was used to generate concentration response curves. Growth IC_50_ (gIC_50_) values correspond to the mid-point of the growth window (between DMSO and T_0_ values).

### Caspase 3/7 Assa

Caspase 3/7 assays were performed as described previously [29]. Caspase-Glo 3/7 readings were normalized to Cell Titer-Glo readings from the same treatment group to correct for differences in cell number. Results were plotted as fold-induction relative to DMSO-treated samples from the corresponding time point.

### siRNA Transfections

Reverse siRNA transfections were performed using Lipofectamine RNAiMAX reagent (Life Technologies, Carlsbad, CA) and 25nM siRNA. All siRNA were from Thermo Scientific (Rockford, IL, USA). Catalog numbers for individual siRNAs are listed in Supplemental Methods. Western blot analysis

Lysates were generated in RIPA buffer (Sigma Aldrich) containing 1x protease and phosphatase inhibitor cocktail (Cell Signaling Technology), following the manufacturer's protocol. Protein concentration was determined via BCA protein assay (Thermo Scientific, Rockford, IL, USA), using BSA as a standard. Equivalent amounts of protein were separated by SDS-PAGE and transferred onto nitrocellulose membranes. Antibodies were diluted in Odyssey blocking buffer (LI-COR Biosciences, Lincoln, NE, USA) containing 0.05% Tween-20 at the manufacturer's recommended dilutions. Images were obtained on an Odyssey infrared imaging system (LI-COR Biosciences).

### Cell Cycle analysis

Propidium iodide staining was performed using the Cycletest Plus DNA reagent kit (BD Biosciences) following the manufacturer's instructions, and samples were analyzed on a FACSCalibur flow cytometer (BD Biosciences). Histograms were generated and cell cycle analysis was performed using FlowJo software (Tree Star, Inc., Ashland, OR, USA).

### Affymetrix Microarray Profiling

Two independent biological replicates of LNCaP, VCaP, PC3, or NCI-H660 cells treated with DMSO, 0.5 µM, or 10 µM of I-BET762 for 24 hours were collected and submitted to Expression Analysis (Durham, NC) for profiling on Affymetrix GeneChip® Human Genome U133 Plus 2.0 arrays. CEL files, corresponding to individual samples, were processed by the Micro Array Suite 5.0 (MAS5) algorithm (http://www.affymetrix.com/support/index.affx), where signal values were scaled to a target intensity of 500 and log_2_ transformed. Differentially expressed probe sets were determined by fitting the data to a linear model and carrying out pair-wise contrasts of treatment versus control. Significant probe sets were filtered for detection based on present/absent calling, an average fold-change >2 or <−2, and *P*-values adjusted for multiple testing correction by false discovery rate (FDR) (Benjamin–Hochberg) < 0.1. Statistical analyses were performed using the limma package from Bioconductor. Significant probes for all treatments and cell lines are provided in Supplemental Table S4.

(http://www.bioconductor.org/).

Functional analyses of gene lists in terms of Gene Ontology Biology Process (GO BP) or pathway enrichment were performed at the gene-level using DAVID (http://david.abcc.ncifcrf.gov/). Gene Set Enrichment Analysis (GSEA) was performed using GenePattern (18, 19). Gene set permutations were used to identify significantly enriched gene sets from the c2 (curated gene sets) and c3 (motif gene sets) MSigDB collections using the signal-to-noise metric for vehicle versus I-BET762-treated samples.

### qRT-PCR Analysis

RNA was purified using the RNEasy Mini kit (Qiagen) and cDNA was generated using the High Capacity cDNA Reverse Transcription kit (Life Technologies), following the manufacturers' instructions. TaqMan analysis was performed on an Applied Biosystems ViiA7 real-time PCR machine, using *GAPDH* as an internal control. Relative expression compared to DMSO was calculated using the Comparative Ct (Ct) method following the manufacturer's protocol (Life Technologies).

### Lentiviral Transduction Experiments

Infections were carried out in serum-free RPMI-1640 media at an M.O.I. of 3 over a period of 24 hours. Media containing virus was then removed and replaced with normal growth media. The following day, transduced cells were selected by the addition of 1 µg/ml puromycin to the growth media. Following 96 hours of selection in puromycin, cells were trypsinized, counted and plated for growth-death analysis, cell cycle analysis, or Western blot in normal growth media containing 0.125 µg/ml puromycin. Assays were carried out as described above.

### *In vivo* Studies

All studies were conducted after review by the Institutional Animal Care and Use Committee at GSK and in accordance with the GSK Policy on the Care, Welfare and Treatment of Laboratory Animals. The Institutional Animal Care and Use Committee at GSK specifically approved these studies. The LuCaP series of prostate cancer xenograft lines was developed by co-author RV at the University of Washington. LuCaP 35CR and LUCaP 145.2 models were passaged and grown in castrated male beige SCID mice (CB17.B6-Prkdc<scid>Lyst<bg>, Charles River Labs). Tumors were measured with calipers and randomized using stratified sampling according to tumor size into treatment groups of 10 mice. I-BET762 was prepared as a solution in 1% methylcellulose vehicle containing 0.2% SDS. I-BET762 in vehicle or vehicle alone was administered orally by individual body weight at 10 ml/kg. Mice were weighed and tumors were measured with calipers twice weekly, and mice were observed daily for any adverse treatment affects. All groups of mice were supplemented with Diet Gel (ClearH_2_O) throughout the study. Mice were euthanized using CO_2_ inhalation according to AVMA guidelines after two consecutive tumor measurements greater than 2500 mm^3^, or if body weight loss greater than 20% was observed. For pharmacodynamic studies, mice were euthanized as described above. Tumors were harvested from euthanized mice and placed in RNAlater (Life Technologies) for RNA isolation as described above.

## Supplementary Tables and Figures






