# Supplementary material for: Inhibition of BET bromodomain proteins as a therapeutic approach in prostate cancer
Source: Oncotarget. 2013 Nov 23;4(12):2419–29. doi: 10.18632/oncotarget.1572 (PMC3926837; doi:10.18632/oncotarget.1572)
Supplement: Supplementary file 1 [file oncotarget-04-2419-s001.pdf]

Inhibition of BET bromodomain proteins as a therapeutic approach in prostate cancer –Wyce et al

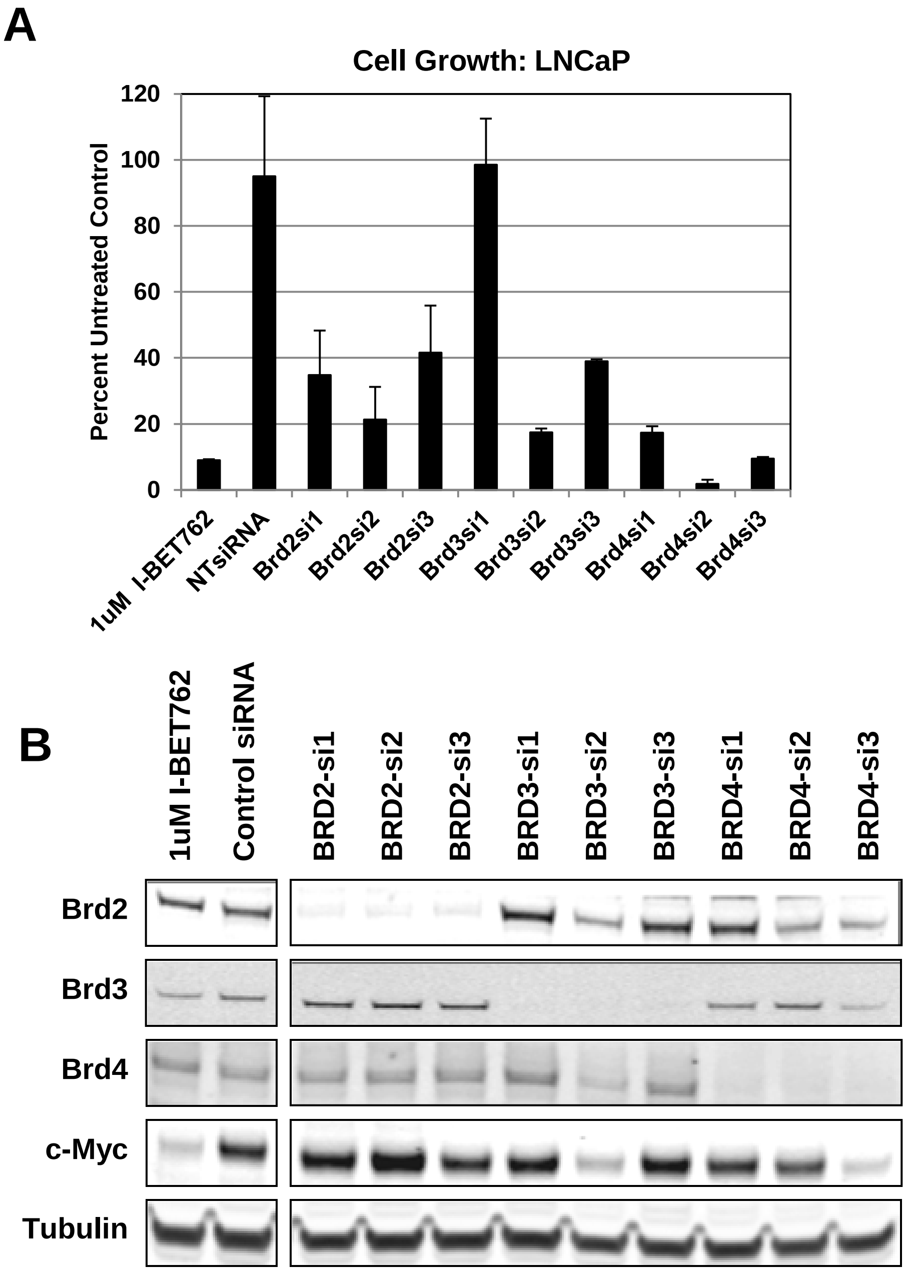

Supplemental Figure S1: Knockdown of *BRD2*, *BRD3*, and *BRD4* in LNCaP cells. A, Analysis of cell proliferation in I-BET762 or siRNA-treated LNCaP cells 6 days post-treatment. Data is presented as percent of untreated control cells, and represents the mean +/- SDM for two independent biological replicates. B, Western blot analysis of BRD2, BRD3, BRD4, and c-Myc expression on day3 post-transfection.

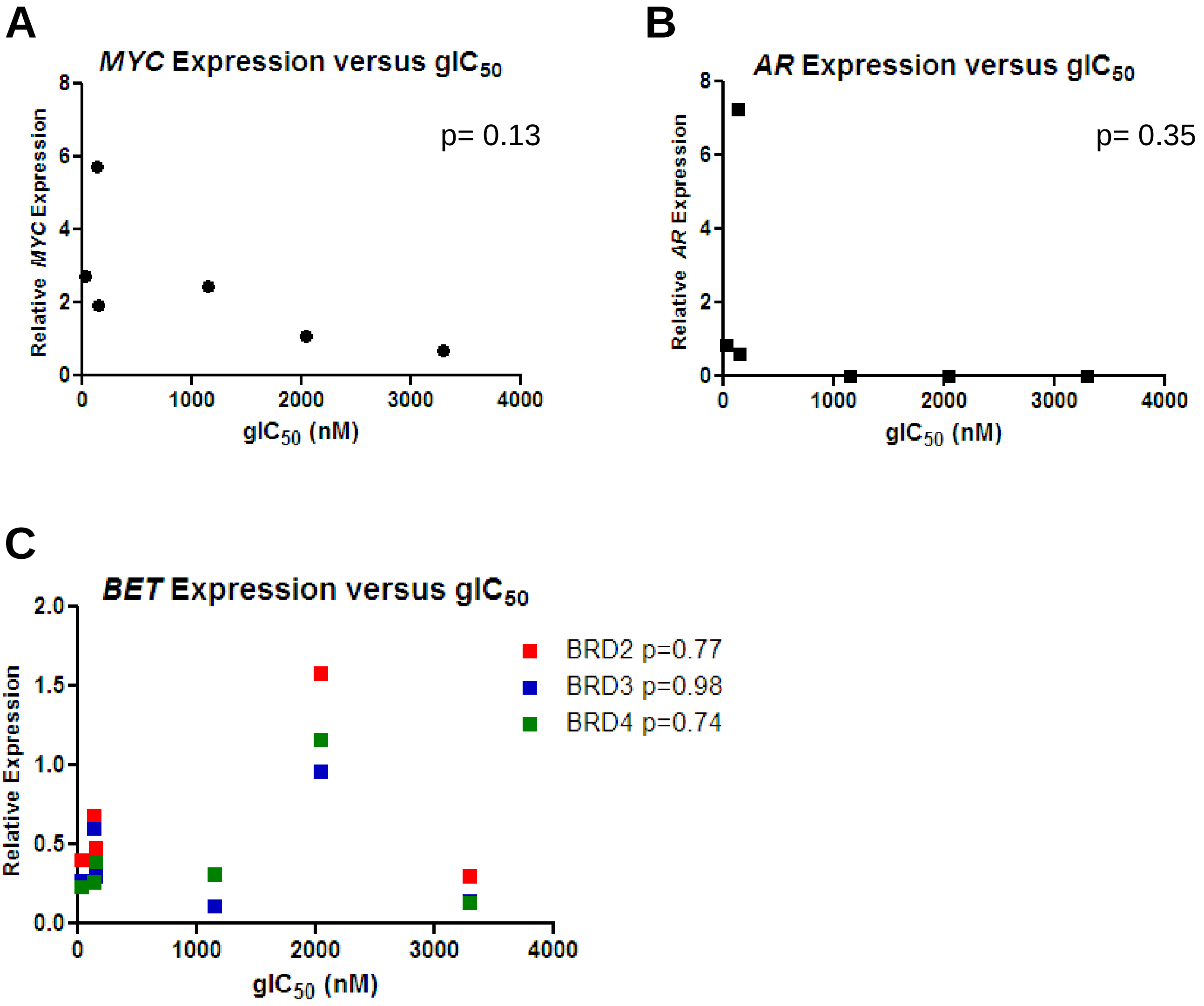

Supplemental Figure S2: Correlation of I-BET762 gIC_{50} to A, *MYC* B, *AR* and C, BET family RNA expression in the prostate cell line panel. P values from two-tailed Pearson correlation analyses are indicated.

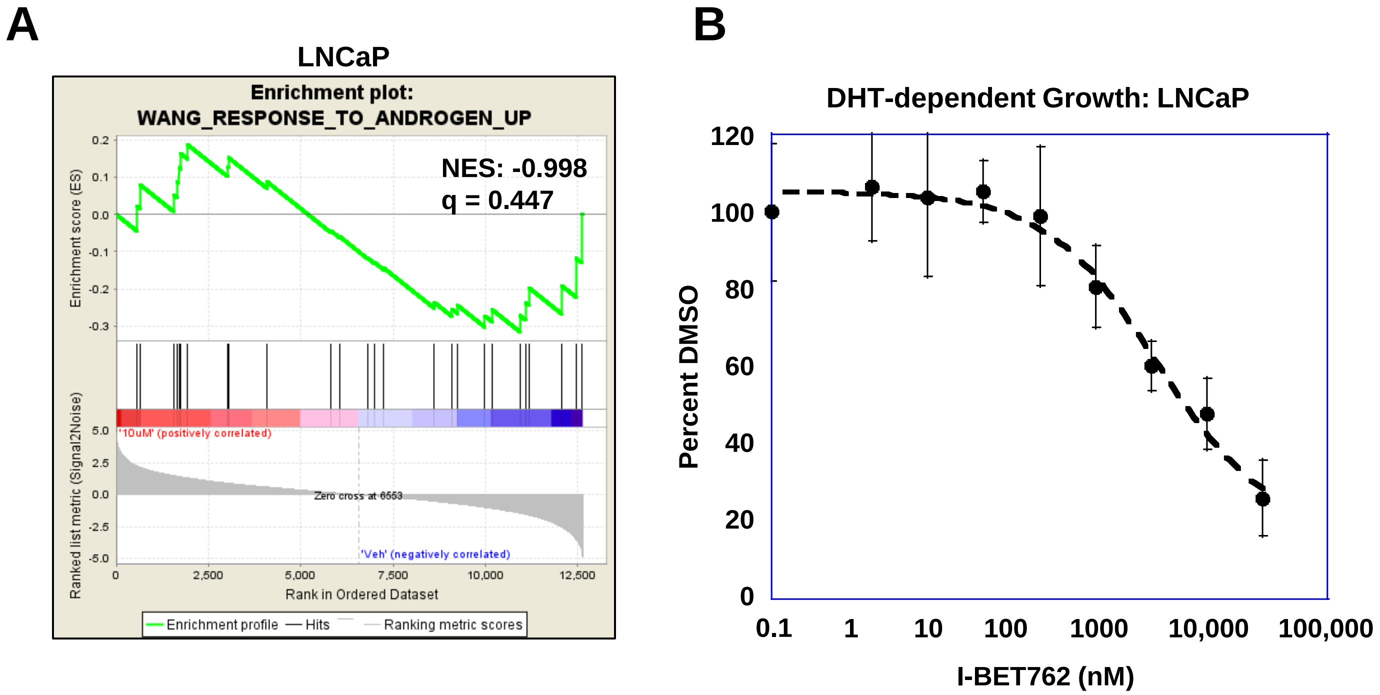

Supplemental Figure S3: Minimal effects on androgen-regulated gene expression or DHT-dependent growth in LNCaP cells treated with I-BET762. A, GSEA enrichment plot showing changes in an androgen-regulated gene signature in LNCaP cells treated with 10 μM I-BET762 for 24 hours. Normalized enrichment score (NES) and FDR q value are indicated. B, Day 6 concentration-response curve for LNCaP cells grown in phenol-red free medium supplemented with 10% charcoal stripped FBS and 10 nM DHT.

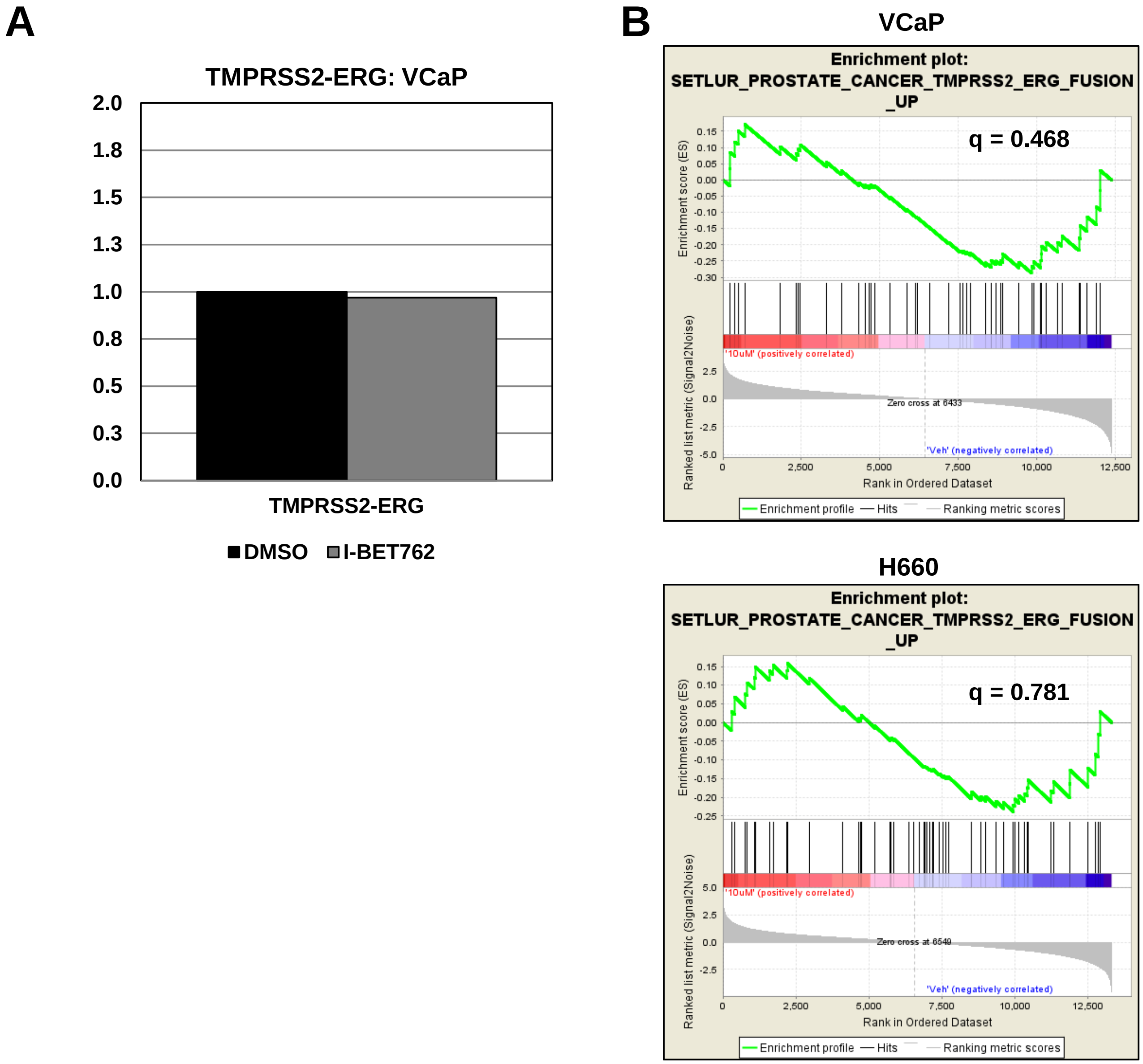

Supplemental Figure S4: TMPRSS2-ERG and its downstream targets are not regulated by I-BET762 treatment. A, qPCR analysis of TMPRSS2-ERG expression in the VCaP cell line following 24 hour treatment with 1 µM I-BET762. B, GSEA enrichment plot showing changes in a TMPRSS2-ERG signature in VCaP and H660 cells treated with 10 µM I-BET762 for 24 hours. FDR q value for each cell line is indicated.

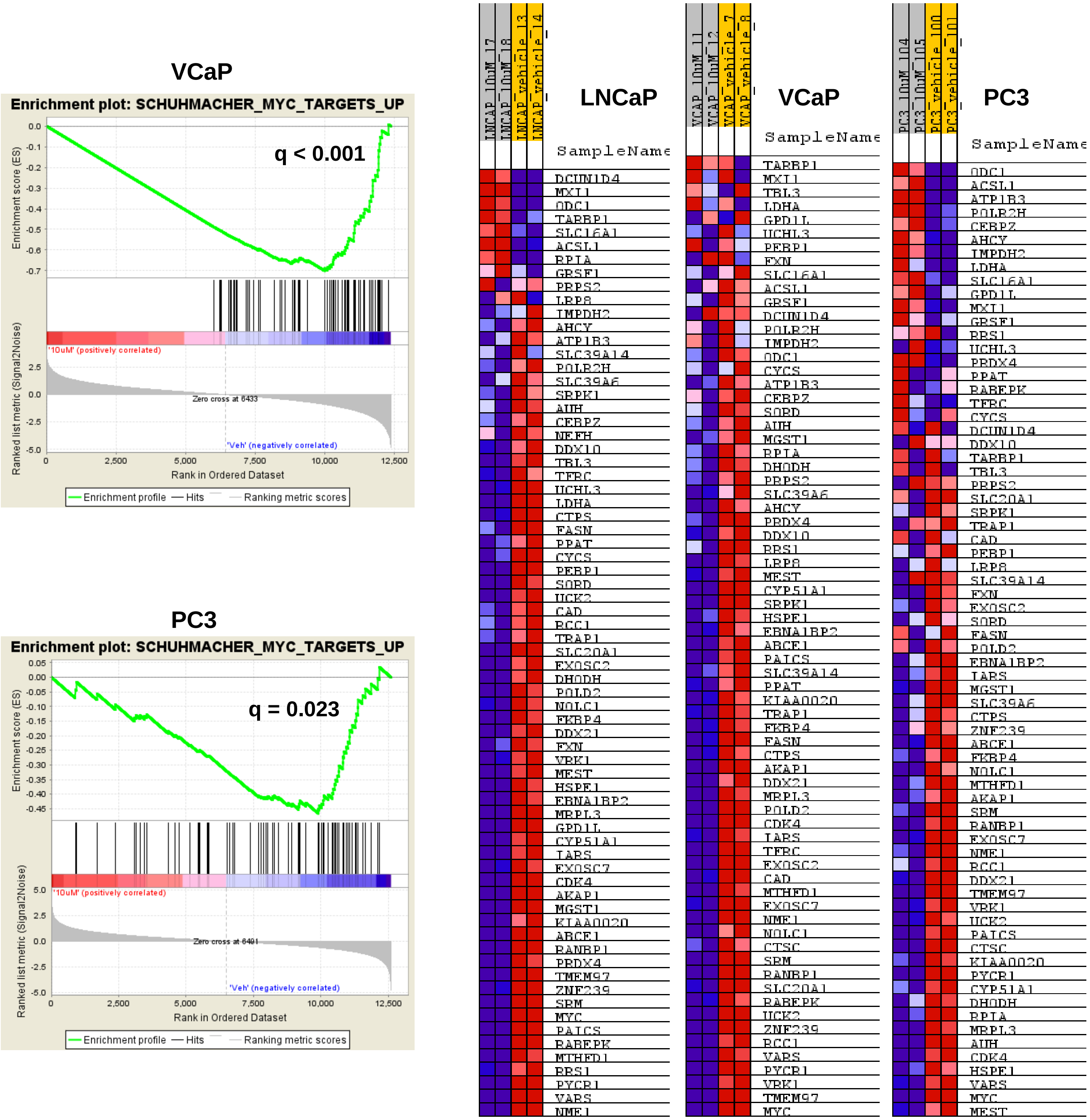

Supplemental Figure S5: GSEA enrichment plots (left) and heat maps (right) depicting gene expression changes associated with the GSEA signature, SCHUHMACHER_MYC_TARGETS_UP, in the indicated prostate cancer cell lines following 24 hour treatment with 10 µM I-BET762.

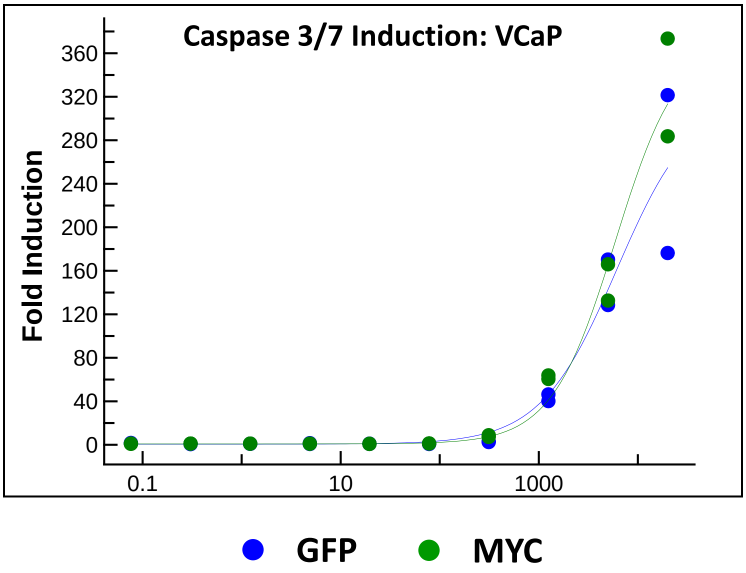

Supplemental Figure S6: Caspase induction in *GFP* or *MYC*-overexpressing VCaP cells following treatment with a titration of I-BET762 for six days. Data is presented as fold induction over DMSO controls, following normalization to total cell number as measured by CellTiter-Glo. Data shown were from a single experiment representative of typical results.

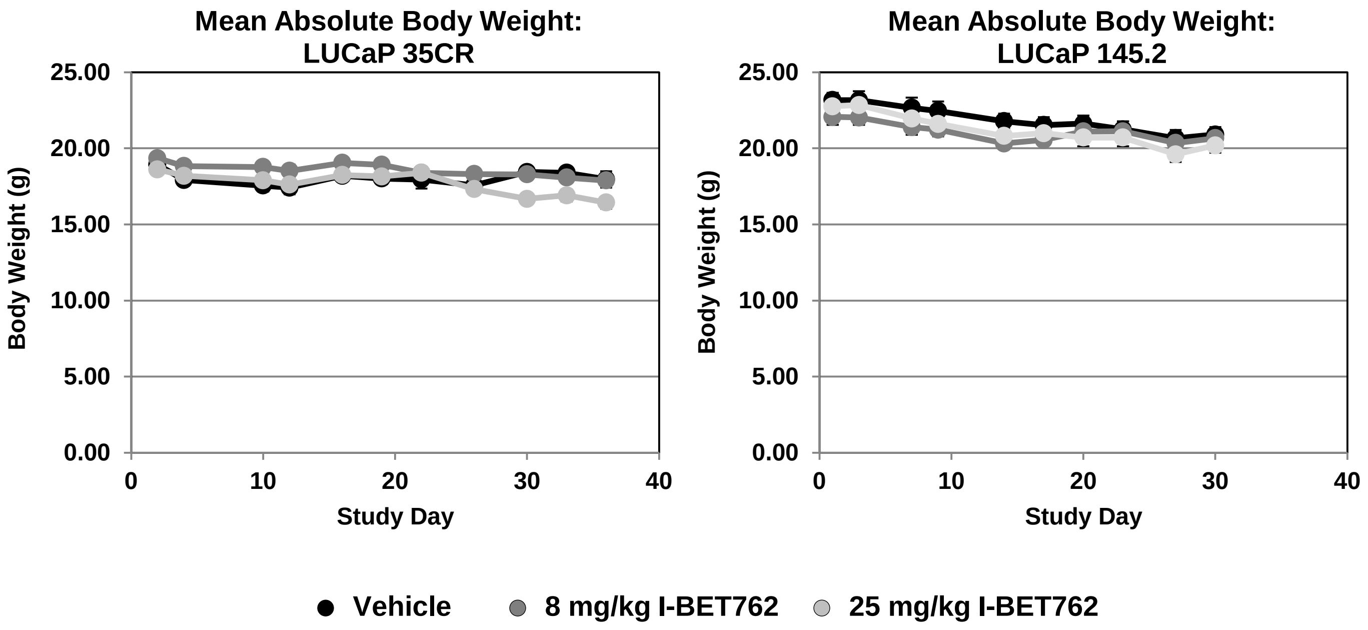

Supplemental Figure S7: Mean absolute body weight for mice in the LUCaP 35CR (left) and LUCaP 145.2 (right) xenograft studies treated with vehicle, 8 mg/kg, or 25 mg/kg I-BET762.
